# Progress towards malaria elimination in Zimbabwe with special reference to the period 2003–2015

**DOI:** 10.1186/s12936-017-1939-0

**Published:** 2017-07-24

**Authors:** Shadreck Sande, Moses Zimba, Joseph Mberikunashe, Andrew Tangwena, Anderson Chimusoro

**Affiliations:** 1 Abt Associates Inc., Block 1 & 2 Westgate, Harare, Zimbabwe; 2 Department of Biological Science, University of Zimbabwe, Harare, Zimbabwe; 3 Ministry of Health and Child Care, National Malaria Control Programme, Harare, Zimbabwe; 4 World Health Organization, Country Office, Harare, Zimbabwe

**Keywords:** Malaria, Malaria vectors, Malaria cases, Malaria control, Malaria elimination

## Abstract

**Background:**

An intensive effort to control malaria in Zimbabwe has produced dramatic reductions in the burden of the disease over the past 13 years. The successes have prompted the Zimbabwe’s National Malaria Control Programme to commit to elimination of malaria. It is critical to analyse the changes in the morbidity trends based on surveillance data, and scrutinize reorientation to strategies for elimination.

**Methods:**

This is a retrospective study of available Ministry of Health surveillance data and programme reports, mostly from 2003 to 2015. Malaria epidemiological data were drawn from the National Health Information System database. Data on available resources, malaria control strategies, morbidity and mortality trends were analysed, and opportunities for Zimbabwe malaria elimination agenda was perused.

**Results:**

With strong government commitment and partner support, the financial gap for malaria programming shrank by 91.4% from about US$13 million in 2012 to US$1 million in 2015. Vector control comprises indoor residual house spraying (IRS) and long-lasting insecticidal nets, and spray coverage increased from 28% in 2003 to 95% in 2015. Population protected by IRS increased also from 20 to 96% for the same period. In 2009, diagnostics improved from clinical to parasitological confirmation either by rapid diagnostic tests or microscopy. Artemisinin-based combination therapy was used to treat malaria following chloroquine resistance in 2000, and sulfadoxine–pyrimethamine in 2004. In 2003, there were 155 malaria cases per 1000 populations reported from all health facilities throughout the country. The following decade witnessed a substantial decline in cases to only 22 per 1000 populations in 2012. A resurgence was reported in 2013 (29/1000) and 2014 (39/1000), thereafter morbidity declined to 29 cases per 1000 populations, only to the same level as in 2013. Overall, morbidity declined by 81% from 2003 to 2015. Inpatient malaria deaths per 100,000 populations doubled in 4 years, from 2/100,000 to 4/100,000 populations in 2012–2015 respectively. Twenty of the 47 moderate to high burdened districts were upgraded from control to malaria pre-elimination between 2012 and 2015.

**Conclusions:**

A significant progress to reduce malaria transmission in Zimbabwe has been made. While a great potential and opportunities to eliminate malaria in the country exist, elimination is not a business as usual approach. Instead, it needs an improved, systematic and new programmatic strategy supported strongly by political will, sustained funding, good leadership, community engagement, and a strong monitoring and evaluation system all year round until the cessation of local transmission.

## Background

A substantial progress in malaria control has been made globally in the past two decades following expanded and intensified efforts. The number of malaria cases worldwide fell by 18%, from about 262 million in 2000 to 214 million in 2015 and mortality decreased by 48%, from 839,000 to 438,000 during the same period [1]. For the first time after the Global Malaria Eradication Programme (GMEP) ceased in the 1970s, a significant reduction in the malaria burden has been documented in the World Health Organization (WHO) African Region [2].

Some countries in the African region have developed malaria elimination plans and are in the process of reorientation for elimination [2]. Malaria elimination does not mean a complete absence of reported malaria cases or a total absence of disease vectors in a geographical area; instead, it refers to an interruption of local transmission (reduction to zero incidence of indigenous cases) of a specific malaria parasite species in a defined geographical area as a result of deliberate activities [3].

Vector control strategies, such as indoor residual spraying (IRS) and use of long-lasting insecticidal nets (LLINs), together with case management (prompt access to diagnosis and effective treatment) are fundamental for reducing malaria transmission [3]. Zimbabwe has a long history of vector control, particularly IRS, dating back to the late 1940s, using benzene hexachloride (BHC), dichloro-diphenyl-trichloro-ethane (DDT), and more recently pyrethroids and organophosphates [4, 5]. Historically in Zimbabwe, use of LLINs has had a much lesser role in malaria control interventions compared to IRS until the first mass campaign in 2010 [6]. Universal coverage by vector control interventions is required for impact and to reduce malaria cases to less than 1 per 1000 populations per annum, which is the level at which elimination should be considered [3]. It is accepted that if a universal coverage of at least 80% use of either IRS or LLINs by populations at risk of contracting malaria is achieved and maintained, malaria burden will be significantly reduced [7]. Large-scale malaria control interventions including application of IRS, deployment of LLINs, intermittent preventive treatment in pregnancy (IPTp), and case management have been conducted with significant successes since the nationwide scale-up activities initiated more than a decade ago [5].

In view of the progress made over the past decade, malaria elimination is being considered as a decisive long-term solution to the serious burden of this disease on a large proportion of the communities in Zimbabwe. The progress made so far appears to outweigh the current challenges such as resistance to insecticides and anti-malarial medicines, outdoor malaria transmission, changes in vector behaviour, re-emergence of vectors, invasion of new areas by vectors, climate change, unpredictable future funding, volatile economic and political situations, intense cross-border population movements, as well as the recent increased inflow of refugees due to the current political unrest in the neighbouring Mozambique.

The aim of the present work is not only to highlight major progress in malaria control, prospects and challenges for malaria elimination, but to contribute also to a better understanding of how Zimbabwe and its partners can ride on the current decline in malaria burden, see feasibilities beyond the present challenges to accomplish malaria control interventions agenda and prepare towards nationwide malaria elimination goals in the near future. This study provides some useful suggestions not only to sustain the current milestones in malaria control interventions, but rather to improve the control strategies for eliminating local malaria transmission in Zimbabwe.

## Methods

### Study sites and populations

**Fig. 1:**
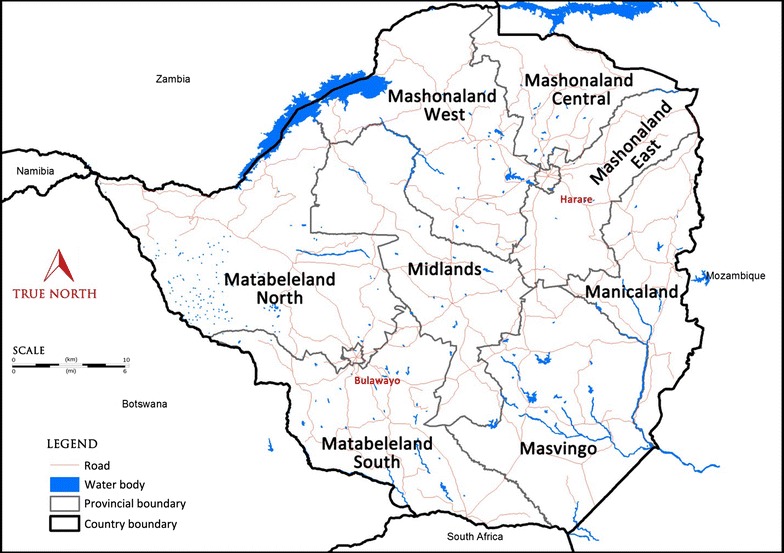
Zimbabwe map showing provincial boundaries and bordering countries

The entirely landlocked nation of Zimbabwe is located between 15° and 22° latitudes, and 25° and 33° longitudes in the southeastern region of Africa, lies wholly within the tropics and borders with four countries (Fig. 1). It has eight rural provinces, and spans an extensive high inland plateau that drops northwards to the Zambezi Valley, bordering with Zambia, and similarly drops southwards to the Limpopo Valley bordering with South Africa, with a total surface area of about 390,757 km^2^. The country has a population of approximately 13 million people whose major economic activities are agro-based [8], with two distinct human settlement patterns. The greater part is the rural set-up, with households that are randomly sited, whereas the smaller portion comprises urban dwellings that have linear housing settlements. In the majority of cases, health centres are strategically located within 10 km-radius of the settlements. The ecological topographies of the country show that it consists primarily of tropical savanna grassland and woodland ecosystem [9]. Despite being located in the tropics, temperate conditions prevail all year round in Zimbabwe, as the climate is moderated by its inland position as well as altitude ranging from 400 to 1200 m above sea level.

The climate can be largely grouped into three seasons: the cold and dry is from May to August, hot and dry from September to October, warm and wet from November to April [9]. Rainfall is seasonal and declines from east to west, with the Eastern Highlands receiving more than 1000 mm annually, while the amount of annual rainfall in the rest of the country varies considerably from 400 to 900 mm. Temperatures on the higher grounds vary from 12 to 13 °C in winter to approximately 24 °C in summer. On the lower lying areas, the temperatures in winter are usually 18–19 °C, and summer temperatures average between 32 and 38 °C, especially in the Zambezi and Limpopo Valleys.

Malaria transmission is seasonal and unstable, causing morbidity and mortality across all age groups [9–11]. Epidemics occasional occur during the warm and wet season, particularly in February to April [5]. The primary vector mosquito species responsible for most of the malaria transmission in Zimbabwe is *Anopheles arabiensis* and *Anopheles funestus* sensu stricto [9, 12–14]. To prevent and control malaria in Zimbabwe, vector control and case management strategies complemented by social behaviour change communication (SBCC) remain the pillars in all regions with moderate to high transmission.

### Data sources

Data were collected through a comprehensive literature review of health systems policies, notified malaria cases and deaths, and programming and elimination reports from the National Malaria Control Programme (NMCP), a Department for Disease Prevention and Control within the MOHCC. Data search was carried out from the records of the WHO, United Nations Children’s Fund (UNICEF), Roll Back Malaria, United States agency for international development/President’s malaria initiative (USAID/PMI), the Global Fund to Fight AIDS, Tuberculosis and Malaria, the National Health Information System 2 (DHIS 2), the NMCP, as well as other published and unpublished reports. Unpublished records were obtained from the DHIS 2, Zimbabwe Malaria Programme Review (ZMPR), Zimbabwe Malaria Indicator Survey (ZMIS), vector control and case management annual reports, and expert opinion from members of the NMCP. Data on malaria morbidity and mortality at the country level from 2003 to 2015 were collected according to the annual submission from health centres to DHIS 2, including province, district, centre name, date of diagnosis, age and gender. Morbidity data comprising either active or passive case detection based on diagnostics using either rapid diagnostic tests (RDTs) or microscopy reported within the study period.

Data on vector control interventions, especially IRS and LLINs were collected using unpublished NMCP annual reports. Indoor residual spraying activities were reported per spray cycle, implemented from September to December annually. Variables obtained from the NMCP’s annual reports included type of insecticide applied, targeted structures for spraying, number of structures sprayed, spray coverage, insecticide used, population protected by IRS, and LLIN distributed by region.

### Data analysis

In this mostly descriptive retrospective study, data were computed into Microsoft Excel, before subjected to statistical analysis using analysis of variance (ANOVA) at 95% confidence limit. The incidence of malaria per 1000 populations was computed per annum, while deaths were by all ages. The data on IRS intervention on malaria cases were aggregated annually from 2003 to 2015 by computing descriptive statistics of the percentage spray coverage and compared to the WHO’s target coverage for impact of ≥80%. For malaria elimination, the WHO framework for malaria elimination was followed [3].

### Ethics approval

Permission to carry out the study was sought and granted by the Zimbabwe’s National Malaria Control Programme Director.

## Results

### Organization of the health system and profile

The health system in Zimbabwe is decentralized, with strong adoption of primary health care concept to deliver health services. The major health services are provided by the hospitals and clinics under the leadership of eight provincial medical directors for the eight rural administrative provinces and two directors for city health services for the two urban provinces. The NMCP which is located in the MOHCC’s head office in Harare, with guidance from five technical sub-committees (case management, vector control, SBCC, procurement and supply chain management, and monitoring and evaluation) is responsible for formulating the national malaria control policies, guidelines, standard operating procedures, and leads operational research. More so, the NMCP monitors the national malaria morbidity and mortality trends, provides technical guidance and training to provinces.

### Resource availability and accessibility

**Fig. 2:**
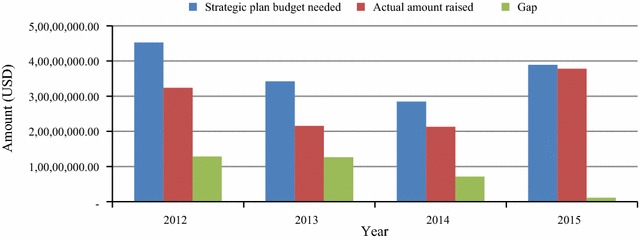
Trends of available funds and gaps for malaria programming in Zimbabwe from 2012 to 2015

Zimbabwe has more than 1780 health facilities strategically located in all the regions of the country. The health institutions are ranked into four tier health system: central, provincial, and district hospitals, as well as clinics/rural health centres (RHCs). All the health facilities are situated within 10 km-radius in villages and urban suburbs to ensure accessibility by the majority of the people. As funding for interventions is one of the most important drivers of success, financial contributions by various agencies from 2007 to 2015 are shown on Table 1. Although there was a notable fluctuation of the programme budget between years for each funding agency, the NMCP’s budget almost trebled in 9 years from 2007 to 2015. The USAID/PMI was consistent for 4 years in its malaria budget allocation for Zimbabwe from 2012 to 2015. With strong government commitment and partners support, the financial gap for malaria programming shrank from about US$13 million in 2012 to US$1 million in 2015 (Fig. 2), achieving a 91.4% (11745425/12853561) funding gap reduction over 4 years.

**Table 1:** Malaria funding agency and their contributions in US dollar from 2007 to 2015

Funder	2007	2008	2009	2010	2011	2012	2013	2014	2015
GoZ	600,000	850,000	1,400,000	1,200,000	1,000,000	1,150,000	1,000,000	950,000	500,000
Global fund	6,800,000	2,100,000	11,320,000	24,500,000	2,600,000	18,988,392	7,505,286	17,576,883	33,425,777
USAID/PMI	–	–	200,000	–	–	12,000,000	12,000,000	12,000,000	12,000,000
WHO	–	–	1,200,000	–	–	–	90,060	42,500	39,000
UNICEF	3,500,000	–	–	–	–	–	–	–	–
DFID	–	–	300,000	–	–	–	–	–	–
EU	3,500,000	–	–	–	–	–	–	–	–
UNDP	–	–	–	–	–	–	–	15,000	–
Private sector	60,000	47,200	60,000	20,000	12,500	80,000	60,000	45,000	22,500
Total	14,460,000	2,997,200	14,480,000	25,720,000	3,612,500	32,218,392	20,655,346	30,629,383	45,987,277

*GoZ* Government of Zimbabwe

### Malaria policies

Following the successful implementation of the Zimbabwe’s National Health Strategy (1997–2007), the National Malaria Strategic Plan (2001–2007) was developed and used until end of December 2007. Between 2008 and 2015, Zimbabwe used National Malaria Control Programme Strategy 2008–2013, which was extended to 2017, and the National Malaria Prevention and Control Policy as major managerial documents for malaria control interventions in all the regions. Various health policies and their major outcome to malaria prevention and control practices are shown on Table 2.

**Table 2:** Health policies and their outcome to malaria control practices in Zimbabwe from 1997 to 2003

Policy	Year	Outcome
Zimbabwe National Health Strategy	1997–2007	Disease prevention and health promotion prioritised
National Malaria Prevention and control policy	2001–2007	In line with the Zimbabwe National Health Strategy, malaria prevention and control as a crucial element to improve health status of the general populations prioritised
National Malaria Control Programme Strategy	2008–2013 extended to 2017	Universal access of malaria control interventions concept, and the global and regional goal for malaria elimination adopted and implemented
Insecticide Treated Net Policy	2003 revised 2006	Only nets with treatment kits were procured and distributed by the Zimbabwe’s NMCP and partners
Zimbabwe Insecticide Nets Implementation Strategy	2009	Only LLINs are being procured and distributed by the Zimbabwe’s NMCP and partners
Spray operator’s manual	2007	Spray operators’ training standardised
Malaria treatment guidelines	2007	All malaria suspected cases parasitological confirmed with either RDT or microscopy before treatment, and free malaria treatment in the government sector implemented
Malaria monitoring and evaluation plan	2008–2013	Implementation of malaria control programmes standardised
Community-based health worker’s policy	2010	Treatment of uncomplicated malaria after parasitological diagnosis using RDTs at village level by community-based health workers was authorised
National Malaria Communication Strategy	2007–2015	Malaria coordination and communication roles centralised to the NMCP

### Vector control

Zimbabwe uses a wide range of vector control tools classified broadly into chemical and non-chemical strategies for controlling either adult or immature forms of malaria vectors, back dating to the late 1940s. The tools are selected on the basis of their efficacy in reducing malaria burden, vector susceptibility status, flora and fauna safety, affordability, cost-effectiveness, and community acceptability. Indoor residual house-spraying which started in 1949 in Zimbabwe is the backbone of vector control interventions, complemented largely by LLINs.

### Entomological surveillance

**Fig. 3:**
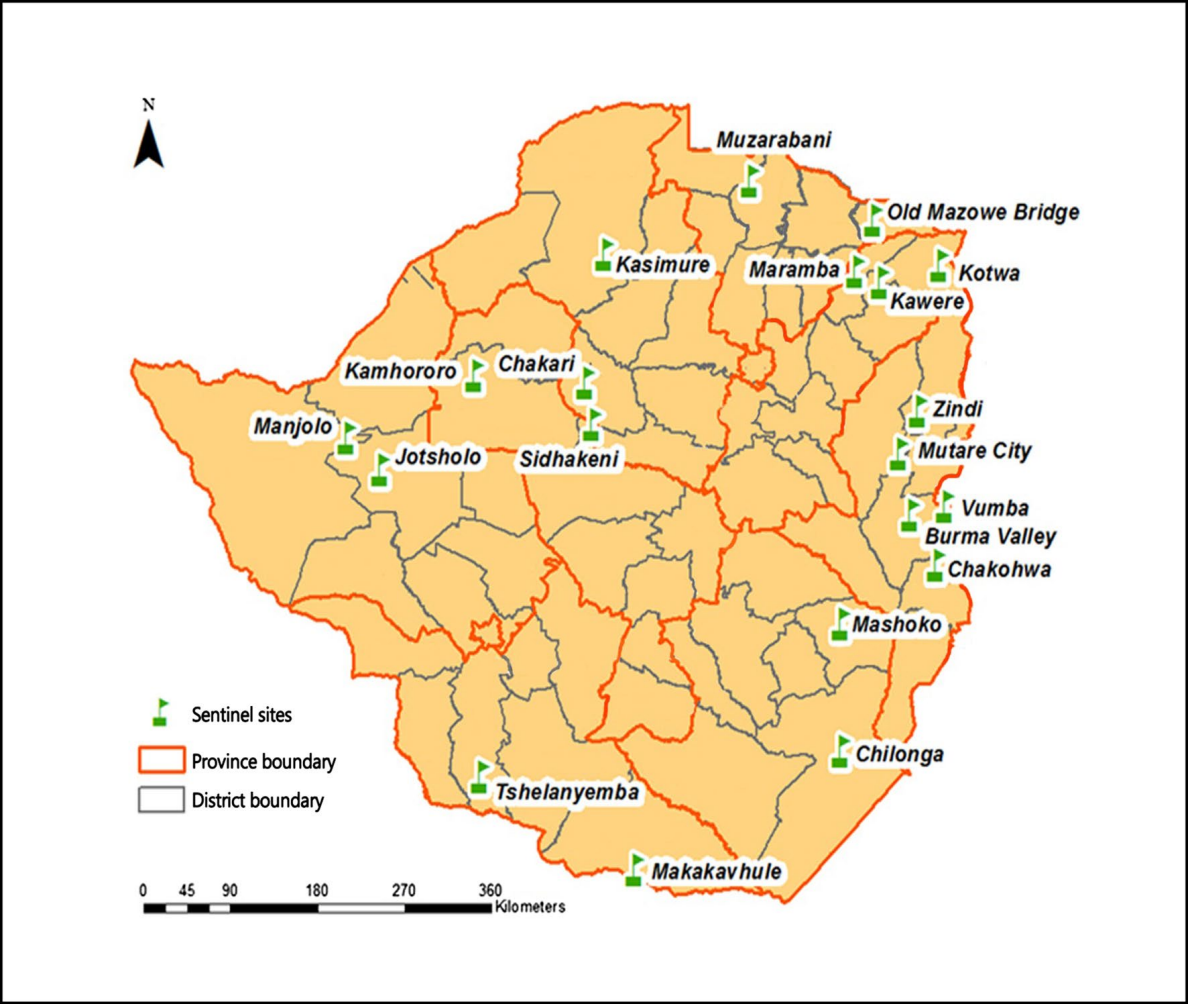
Zimbabwe map showing names and locations of sentinel sites established in the period 2010–2015

The entomological surveillance activities were conducted throughout the period 2003–2015, but intensified from 2010 through to 2015 following the establishment of 20 sentinel sites (Fig. 3). Mosquitoes are mostly sampled using pyrethrum spray catches (PSC), prokopac battery powered aspirator, Centers for Disease Control and Prevention (CDC) light traps, window traps and larval collection methods. Susceptibility assays are used to detect evidence of insecticide resistance, and the results are used in planning for IRS activities, particularly selection of insecticide for indoor house spraying. Between 2005 and 2008, two studies in Gokwe south district of Zimbabwe reported DDT and permethrin resistance in *An. arabiensis* [12, 15]. Similarly, in 2014 and 2015, susceptibility assays in Mutare and Mutasa districts of Manicaland Province in Zimbabwe revealed high levels of resistance to pyrethroids and carbamates in *An. funestus*, but were susceptible to both DDT (organochlorine) and pirimiphos-methyl (organophosphate) [13, 16]. In 2015, studies in Binga, Beit Bridge, Hurungwe, and Sanyati districts reported resistance to lambda-cyhalothrin and bendiocarb in the *Anopheles gambiae* complex, and possible resistance to DDT and etofenprox (NMCP report, unpublished data). However, the observed possible resistance was not confirmed as recommended by the WHO [17].

Results of the WHO cone bioassays conducted on pirimiphos-methyl-sprayed surfaces in Mutare and Chimanimani districts, Zimbabwe in 2014 and 2015 showed 100% mortality 24–48 h post-spray. Insecticide decay was observed as from the 3rd month post spray in both districts. The longevity of insecticide on the sprayed surfaces was 4 and 5 months in Mutare and Chimanimani respectively (The NMCP report, unpublished data). Elsewhere in the country cone bioassay results were insufficient and in most cases inconsistent and not well documented for a meaningful analysis.

### Indoor residual house spraying

House spraying is one cycle per annum, commencing in September through to December of the same year, targeting at most 47 moderate to high malaria burdened districts. Results of indoor residual house spraying clearly showed a continued upward trend for both room coverage and population protected (Table 3). Percentage coverage for IRS and population protected increased steadily from 2003 to 2006, and declined sharply in 2007, before a gradual rise from 2008 to 2015. The population protected target of 95% set by the NMCP was achieved only once, while the WHO target of at least 80% was accomplished 9 times over 13 years. The relation between target rooms and populations varied greatly between years, especially during the period 2005–2009.

**Table 3:** Indoor residual house spraying coverage and population protected in Zimbabwe from 2003 to 2015

Year	Target rooms	Rooms sprayed	% coverage	Target pop.	Pop. protected	% pop. protected
2003	2,235,151	625,842	28	4,732,872	946,574	20
2004	2,175,026	1,350,403	62	3,373,034	2,031,509	60
2005	1,839,727	1,271,474	69	1,875,472	1,608,848	86
2006	1,764,368	1,212,572	69	2,920,561	1,659,393	57
2007	1,413,074	588,994	42	2,436,172	742,289	30
2008	1,111,663	958,045	85	1,630,915	1,304,732	80
2009	1,992,181	1,638,303	86	3,096,049	2,662,602	86
2010	2,255,318	2,023,159	90	3,478,413	3,095,788	89
2011	2,423,091	2,253,474	93	3,496,756	3,217,016	92
2012	2,420,141	2,178,127	90	3,135,886	2,728,221	87
2013	2,512,127	2,286,034	91	3,608,898	3,248,008	90
2014	2,619,334	2,383,594	91	3,823,787	3,517,884	92
2015	2,353,461	2,235,787	95	3,371,473	3,236,614	96

### Long-lasting insecticidal nets

The first free mass LLIN distribution was implemented in 2010 targeting one net per sleeping space or per two people in 30 districts with moderate to high malaria burden, and the same districts benefited also from IRS. Overall, ownership of at least one net per household ranged from 23% in 2008 to 74% in 2014. Although data on bed net utilization were not clearly documented, the estimates on general population who slept under nets the previous night increased from 14.5% in 2008 to 57.9% in 2012, and 58.8% in 2014 (ZMIS, unpublished data).

### Therapeutic efficacy testing

Zimbabwe has a total of eight functional therapeutic efficacy testing (TET) sentinel sites, equitably distributed throughout the eight rural provinces in the country. Therapeutic efficacy testing activities are conducted every 2 years by Zimbabwe’s National Institute of Health Research (NIHR) to monitor in vivo efficacy of anti-malarial medicines used to treat malaria in the country. The monitoring exercise follows the WHO [18] protocol, with modification to include children >5 years old. Until late 1990s, chloroquine (CQ) was the first-line medicine to treat uncomplicated malaria in Zimbabwe. Chloroquine was then replaced in 2000 by a free combination of CQ and sulfadoxine–pyrimethamine (SP) following confirmation of CQ resistance recorded at all eight TET sites. Similarly, in 2004, studies at all eight sites indicated treatment failure of a combination of CQ and SP, resulting in the recommendation for its replacement with artemisinin-based combination therapy (ACT). In 2010 and 2014, the efficacy of ACT at all the eight sites was 96 and 97.5%, respectively, in the treatment of uncomplicated *P. falciparum* malaria; consequently, its use was maintained.

### Malaria diagnosis

Prior to 2004, malaria diagnosis in Zimbabwe was either by clinical or parasitological confirmation using microscopy. Following the revision of malaria policy and the introduction of RDTs in 2004, the country adopted the concept of parasitological diagnosis of all suspected malaria cases using either RDTs or microscopy, although the fully implementation of this policy was delayed for 5 years due to inadequate financial support, especially to procure sufficient RDT kits for the entire nation. While malaria policy emphasizes parasitological confirmation by RDTs or microscopy of all suspected malaria cases, this policy is not followed in confirmed malaria outbreaks or when RDTs ran out of stock at peripheral health facilities without microscopy. Percentage stock out levels of RDTs (calculated as the proportion of health facilities that report non availability of RDTs for more than 1 week in 3 months) varied in each quarter and year, with peak during the first quarter of 2014 (Table 4).

**Table 4:** Percentage RDT stock out levels in Zimbabwe from 2012 to 2015

Year	% RDT stock out levels			
	Q1	Q2	Q3	Q4
2012	19.2	9.3	5.0	9.0
2013	12.4	5.0	7.0	ND
2014	24.3	17	8.0	4
2015	8.0	9.0	ND	ND

*Q1* quarter 1, *Q2* quarter 2, *Q3* quarter 3, *Q4* quarter 4, *ND* no data

### Malaria treatment regimens

While the malaria diagnosis and case management policy changed to the use of ACT in 2004, implementation commenced at a larger scale in 2007. Between 2004 and 2007, an interim treatment policy was CQ plus SP. Since 2007, the policy has been ACT as first line to treat uncomplicated malaria as outpatients, followed by oral quinine plus doxycycline or clindamycin as a 2nd line treatment. Severe malaria cases are managed as in-patients with parenteral quinine plus doxycycline or clindamycin, including the treatment of any other complication that would have arisen during the course of treatment. Even though the national policy is to ensure that there are no stock outs of malaria medicines in all health centres, the country experienced relatively minor ACT stock-outs (calculated as the proportion of health facilities that report non availability of ACT for more than 1 week in 3 months) from 2012 through to 2015 (Table 5). Stock-out of ACT was below 10% for most of the quarters, save for the third quarter of 2013 when it was above 20%.

**Table 5:** Percentage ACT stock out levels in Zimbabwe from 2012 to 2015

Year	% ACT stock out levels			
	Q1	Q2	Q3	Q4
2012	4.0	6.2	1.0	3.0
2013	5.5	0.9	23.7	ND
2014	3.2	2.0	1.0	0.0
2015	3.0	4.0	ND	ND

*Q1* quarter 1, *Q2* quarter 2, *Q3* quarter 3, *Q4* quarter 4, *ND* no data

### Community-based management of malaria

In 2010, the community-based health workers (CBHW) policy was approved by the malaria case management technical subcommittee, with support from the Ministry of Health’s Directorate of laboratory services. During the same year, CBHW was piloted in Zimbabwe’s Mashonaland West Province. Ninety CBHWs were selected from the villages and trained to diagnose malaria using RDTs and treat all positive uncomplicated cases with ACT in the villages they reside. Following the success of the programme, the pilot project was expanded to cover most villages of the eight rural provinces, with more than 1000 CBHWs trained and dispensing ACT to RDT-malaria positive patients by 2011. After training, each CBHW was issued with a bicycle for domiciliary visits and a malaria testing and treatment kit. The malaria kit is replenished at the end of each month or when the commodities reach minimum stock level (1 month’s supply) whichever comes first.

### Malaria in pregnancy

The intermittent preventive treatment in pregnancy (IPTp) using SP to reduce maternal and neonatal morbidity and mortality was recommended by case management technical subcommittee and approved by MOHCC in 2004. The strategy aimed to achieve a coverage of at least 85% of pregnant women attending antenatal care in the 30 moderate to high malaria burdened districts with at least two doses of IPTp, except those on co-trimoxazole prophylaxis. Three doses of SP are given to pregnant women, the first dose at 16 weeks or after quickening, the second at 26–28 weeks, and the third at 34–36 weeks of gestation and administered as directly observed treatment. However, there were uncertainties in the data provided on IPTp and in most cases insufficient or missing that no meaningful analysis could be made.

From 2009, the 1st line treatment for malaria in pregnancy before 16 weeks has been oral quinine 600 mg every 8 h for 7 days with clindamycin 300 mg every 8 h for 7 days, and ACT is administered after 16 weeks. For severe malaria, parenteral quinine starting with a loading dose is the medicine of choice.

### Trends in malaria burden

**Fig. 4:**
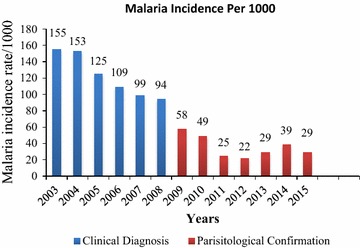
Trends of malaria incidence per 1000 populations in Zimbabwe from 2003 to 2015

**Fig. 5:**
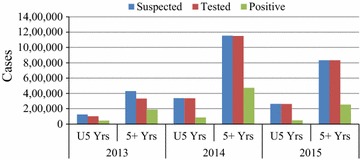
Distribution of malaria cases by age in Zimbabwe, 2013–2015

There has been a decline in malaria incidence over the past decade from the era of mostly clinical diagnosis to the period of parasitological confirmation by either RDTs or microscopy (Fig. 4). From 2012, the cases increased relatively sharply for two consecutive years before dropping again in 2015. Overall, malaria cases reported declined by 81% (126/155) from 2003 to 2015, and the disease occurred in all age groups. Positivity rate decreased from 42% in 2013 to 28% in 2015 in all age groups (Fig. 5). In children <5 years old the malaria positivity rate more or less followed the same trend, declining from 35 to 18% for the same period.

### Malaria deaths

**Fig. 6:**
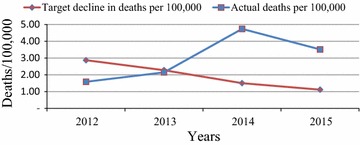
Trends of inpatient malaria deaths in Zimbabwe from 2012 to 2015

A total of 1594 deaths attributed to malaria were recorded between 2012 and 2015. The annual number of deaths associated with malaria increased by approximately 100% from 207 in 2012 to 473 in 2015. Similarly, inpatient malaria deaths per 100,000 populations doubled in 4 years, from almost 2/100,000 in 2012 to 4/100,000 populations in 2015, exceeding the yearly national target decline in mortality per 100,000 populations in Zimbabwe (Fig. 6). The case fatality rate fluctuated over the 4 years (2012–2015) ranging from 0.05 to 0.11%, highest during the 2015 (0.11%) malaria season. Overall, the number of people who died of malaria per 100,000 populations was less than the NMCP’s target mortality decline in 2012 and 2013, but surpassed in 2014 and 2015.

### Malaria elimination

**Fig. 7:**
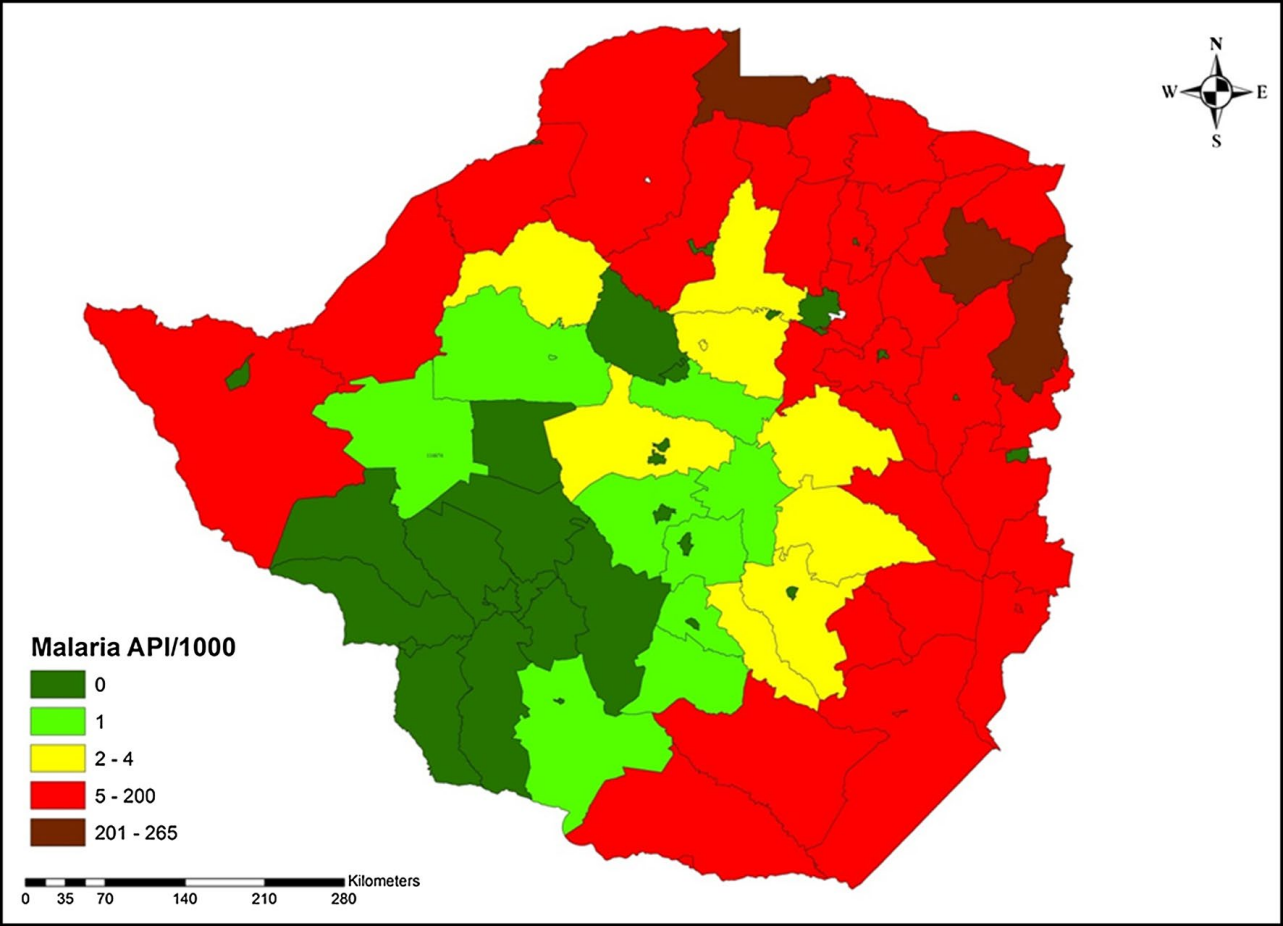
Zimbabwe map showing annual parasite incidence per 1000 populations in 2015

Following a decline in malaria morbidity in Zimbabwe over the past decade, the country adopted the global and regional agenda for malaria elimination by 2030 [19]. To achieve this agenda, Zimbabwe adopted a five phase programme for malaria control interventions and elimination. Phase 1 was meant for malaria control in areas with annual parasite incidence (API) above 200 per 1000 populations, phase 2 was for consolidation of malaria control zones with API between 5 and 200, phase 3 for malaria pre-elimination in areas with API >1 but <5, phase 4 malaria elimination in areas with API ≤1, and phase 5 included malaria free zones with zero local cases of malaria (Fig. 7). Malaria control interventions are tailored according to programme phases informed by epidemiological and entomological data. In Zimbabwe, the concept of malaria elimination was widely discussed and agreed upon by the NMCP and partners as far back as 2010. In 2012, following the reduction in malaria cases to API <5 per 1000 populations in most of the seven districts of Matabeleland South Province, the province was epidemiologically identified by the government of Zimbabwe’s Ministry of Health as the focus of malaria infections that could be eliminated first through reorientation of malaria control and prevention programmes. Subsequently, in 2015, API of 13 districts in Midlands, Matabeleland North, and Mashonaland West Provinces declined to <2 per 1000 populations. Of these districts, seven are in Midlands, five in Matabeleland and one in Mashonaland West. The 13 districts were upgraded in 2015 for programme reorientation by changing set of interventions, aggregating to 20 districts including seven that pioneered the implementation of malaria pre-elimination activities since 2012. Elsewhere in the country, the districts continue to implement malaria control interventions. To strengthen the move from malaria control to elimination, by 2015, Zimbabwe had joined four important regional malaria elimination networks. These alliances include the E 8 countries (Angola, Botswana Mozambique, Namibia, South Africa Swaziland, Zambia and Zimbabwe), the ZAMZIM (Zambia and Zimbabwe), the MOZAZI (Mozambique, Zambia and Zimbabwe), and the MOZIZA (Mozambique, Zimbabwe and South Africa). While these associations are reported by the NMCP to be fully functional, data on the number and frequency of meetings held in-country or across borders by each group over the years could not be accessed for analysis.

## Discussion

Malaria could be eliminated in Zimbabwe as it has been in Europe and Americas, as well as a few countries in Africa [20]. The consistent decline in malaria burden over the past 13 years in Zimbabwe illustrates a clear roadmap towards malaria elimination in the near future. Malaria transmission decreased by 81% from 2003 to 2015. Similar reduction in the malaria burden has been reported recently in other countries of sub-Saharan Africa [21]. Malaria control efforts have reduced malaria burden to levels where elimination is a possibility, primarily through sustained provision of universal access to vector control interventions, especially IRS and LLINs, as well as early diagnosis and treatment with effective anti-malarials [3, 22].

The findings of this study clearly showed that vector control is an essential component of malaria control and elimination. Indoor house spraying has a long history in Zimbabwe back-dating to the 1940s [4]. Although results of this work showed that spray coverage and population protected were relatively low in the early 2000s, the coverage increased drastically to 95 and 96% respectively in 2015, suggesting that the majority of the people residing in high risk malaria areas were protected through the application of IRS. The elimination of malaria transmission in some temperate regions around the globe during the eradication era in the 1950s and 1960s was predominantly IRS-based using mostly DDT [23], demonstrating its programmatic effectiveness in malaria control and elimination. A study conducted in Uganda to evaluate the impact of IRS using lambda-cyhalothrin on malaria morbidity showed a consistent decline in number of cases in the first 4 months post spray [24]. More recently, work by Kanyangarara et al. [25] showed that application of IRS using pirimiphos-methyl had a significant impact on malaria incidence in Mutasa district, Zimbabwe. Despite the increase in room and population protected coverage over the years, the substantial variations in the relation between target rooms and population between years observed in this study are worrisome. Perhaps the variations are due to use of different data sources each year to calculate room and population coverage.

While IRS has been applied for over six decades in Zimbabwe, mosquito nets were little distributed until recently when free mass campaign was introduced in 2010. Mosquito nets provide a physical barrier against mosquitoes; also, due to insecticide impregnated in them, they reduce the longevity of mosquito populations, decreasing the chance of malaria transmission [26]. To achieve, consolidate and maintain universal coverage of equal or greater than 80% use of LLINs by populations at risk of malaria is fundamental for the reduction of the disease burden [27]. Findings from the present work appears to be way off this target, with the estimates on general population who slept under nets the previous night ranging from 14.5% in 2008 to 58.8% in 2014 (ZMIS, unpublished data). However, these results are consistent with those observed in western Kenya highlands which showed ITN usage to be 28–49% [28], compromising the impact of nets on the disease burden. In Zimbabwe, it appears there are no studies on the impact of bed nets on malaria burden to the general populations at risk.

The substantial impact of insecticide-based vector control on malaria burden is dependent upon susceptibility status of malaria vectors. The present work has reported with concern the resistance in mosquito populations to pyrethroids and carbamates in Mutare, Mutasa and Gokwe districts of Zimbabwe [12, 13, 15, 16], threatening the effectiveness of insecticide-based interventions such as LLINs and IRS. Implication of pyrethroid resistance in the reduction of LLIN effectiveness was reported in West Africa [29–31], while that of IRS was recently reported in Mutasa district, Zimbabwe [25]. It is, therefore, important to regularly monitor the bionomics of malaria vectors for evidence-based vector control interventions. Although the establishment of 20 sentinel sites documented in this study is helpful for evidence-based decision making by the NMCP and partners to control and eliminate malaria, the number of sites appears to be comparably too low. Adequate number of sentinel sites that will consistently sample the target mosquito populations over time is critical. The choice and number of sites should be based on advice from the technical committees, programme partners, and recommendations for insecticide resistance monitoring.

Although the increase in IRS coverage might have significantly contributed to malaria decline, other factors such as use of RDTs, effective treatment, management of malaria in pregnancy, community-based management of malaria, increased funding, and strong government commitment might have also played a role in the visible malaria reduction in Zimbabwe. A consistent and adequate annual budget is an important driver for malaria control and elimination. Observations of the present work clearly showed the trebling of the NMCP’s budget for malaria programming in 9 years from 2007 to 2015, as well as shrinking of the financial gap, achieving a 91.4% funding gap reduction over 4 years from 2012 to 2015. In addition to budget rise, there has been equally substantial increase in the number of health facilities and personnel. Presumably, the setting might have paved the way for early treatment-seeking behaviour by the majority of the population at risk, as well as better management of patients by health staff.

The Zimbabwe’s malaria case management campaign produced also major contribution. This was the establishment of eight TET sentinel sites to evaluate efficacy of first line anti-malarials, which resulted in the introduction of ACT to combat CQ and SP-resistant *P. falciparum*. In addition, the roll out of RDTs in all health facilities and the community management of malaria campaign were fundamental for a rapid diagnosis and early treatment of malaria cases, contributing significantly to the reduction of the disease burden shown in the results of the present study. Although morbidity showed a downward trend over the years, malaria-related deaths per 100,000 populations almost doubled from 2012 to 2015. Reasons for increases in malaria deaths were not clear. Presumably, the increase in the number of malaria-related deaths were due to lack of quality of care for patients, lack of knowledge for management of severe malaria in low transmission areas or at referral centres, and non-use or lack of knowledge to use intravenous and rectal artesunate in severe cases. Reports from Mpumalanga Province in South Africa on cases and inpatient malaria deaths painted a different picture from the findings of this study, with mortality declining by 85% between 2001 and 2009 [32].

Whilst the results of the present work showed progress towards malaria elimination, it has been observed with concern that there were some few instances when data were incomplete or missing. Clear examples are data for malaria in pregnancy (MIP) and LLINs. The explanation for missing or incomplete data for MIP could be associated with late booking of antenatal care (ANC) by pregnant women, erratic supplies for SP at health facilities, and to a lesser extent, improper and inconsistent recording, resulting in insufficient data that no reliable analysis could be made. Similarly, incorrect and inconsistent documentation could also explain the missing data for LLINs. Consistent and timely distribution of commodities, strong community involvement, and proper recording of data always are critical for malaria control and elimination.

## Conclusions

Given the substantial progress made to reduce the trends of malaria burden by 81% from 2003 to 2015, the Zimbabwe’s NMCP took the right decision to upgrade 20 districts to reorient interventions from control to pre-elimination guided by the WHO’s continuum and framework for malaria elimination. While it appears certain that opportunities exist in Zimbabwe to accomplish the global goal for malaria elimination, it would be feasible if the new technical and operational challenges to the permanent interruption of local transmission could be overcome. The occurrence of resistance to malaria medicines and insecticides, a genuine possibility of reduced funding by government and partners due to exceptionally low incidence rates, improper guidance to the circumstances under which it may be appropriate to scale-back vector control interventions, and failure to strengthen surveillance systems, as well as lack of data for evidence-based decision-making are the new challenges threatening to reverse the gains made towards malaria control and elimination in Zimbabwe.

Great potential and opportunities to eliminate malaria in the country exist. If malaria was eliminated in temperate regions of Europe and Americas, and other few African countries, applying consistently the same strategies with evidence-based modifications, the disease could be eliminated also in Zimbabwe. Even if evidence suggests that current control interventions have played a greater part to substantially reduced malaria burden, elimination is not a business as usual approach. Instead, it needs an improved, systematic and new programmatic strategy supported strongly by political will and sustained funding. More so, to achieve malaria elimination goal, the Zimbabwe’s NMCP requires good leadership, mentoring, skill building and use, community engagement and training, as well as operational research, and strong monitoring and evaluation system all year round until the cessation of local transmission in the country.

## Abbreviations

ACT: artemisinin-based combination therapy
API: annual parasite incidence
BHC: benzene hexachloride
CBHW: community-based health worker
CDC: Centers for Disease Control and Prevention
CQ: chloroquine
DDT: dichloro-diphenyl-trichloro-ethane
DFID: Department for International Development
DHIS 2: District Health Information System two
E8: elimination eight
EU: European Union
GMEP: Global Malaria Eradication Programme
GoZ: Government of Zimbabwe
IPTp: intermittent preventive treatment in pregnancy
IRS: indoor residual spraying
ITN: insecticide-treated net
LLINs: long-lasting insecticidal nets
LSM: Larval source management
MIP: malaria in pregnancy
MOHCC: Ministry of Health and Child Care
NIHR: National Institute of Health Research
NMCP: National Malaria Control Programme
PSC: pyrethrum spray catch
RDT: rapid diagnostic kits
RHC: rural health centre
SBCC: social behaviour change communication
SP: sulfadoxine–pyrimethamine
TET: therapeutic efficacy testing
UNDP: United Nations Development Programme
UNICEF: United Nations Children’s Fund
WHO: World Health Organization
ZMIS: Zimbabwe Malaria Indicator Survey
ZMPR: Zimbabwe Malaria Programme Review
